# Effects of pleasant sound on overnight sleep condition: A crossover randomized study

**DOI:** 10.3389/frsle.2022.986333

**Published:** 2022-10-18

**Authors:** Shota Saeda, Koichi Fujiwara, Takafumi Kinoshita, Yukiyoshi Sumi, Masahiro Matsuo, Kiyoshi Yamaki, Takahiro Kawashima, Hiroshi Kadotani

**Affiliations:** ^1^Department of Material Process Engineering, Nagoya University, Nagoya, Japan; ^2^Department of Systems Science, Kyoto University, Kyoto, Japan; ^3^Department of Psychiatry, Shiga University of Medical Science, Otsu, Japan; ^4^Division of Electronic Device, Yamaha Corporation, Shizuoka, Japan; ^5^Division of Audio Equipment, Yamaha Corporation, Shizuoka, Japan

**Keywords:** pleasant sound, polysomnography, sleep spindle, EEG analysis, automatic spindle detection algorithm

## Abstract

It is desirable to improve sleep quality since poor sleep results in decreases in work productivity and increases in risks of lifestyle-related diseases. Sleep spindles in sleep EEG are waveforms that characterize non-REM sleep Stage 2 (Stage N2). Music therapy has been adopted as a non-pharmacological therapy for sleep quality improvement; however, few studies mention the relationship between music during sleep and spindles. We conducted a crossover randomized study to investigate music's effects on spindles and sleep parameters. Polysomnography (PSG) was performed on 12 adult males with sleep difficulties over three nights, during which they were exposed to three different acoustic environments–silent, white noise, and pleasant sounds–throughout the night, in a crossover randomized setting. Half of the participants with large WASO were defined as the sleep maintenance difficulty group. We investigated whether pleasant sounds shortened sleep onset latency (SOL) and increased the number of spindles (SN) and spindle density (SD) compared to white noise, using silent as the reference. The spindles were detected using the previously reported automatic spindle detection algorithm. After one patient was excluded due to data corruption, a total of 11 participants, including the sleep maintenance difficulty group (*n* = 5), were analyzed. For all participants, SOL was not significantly shorter with pleasant sound than with white noise (p = 0.683); for the sleep maintenance difficulty group, SOL tended to be shorter with pleasant sound than with white noise (p = 0.060). Compared to white noise, the SN increased in pleasant sound for 7 of 11 (4 of 5 in the sleep maintenance difficulty group), and SD increased for 5 of 11 (3 of 5 in the sleep maintenance difficulty group). The results suggest that all-night background sound exposure may affect SN and SD. Future research should investigate whether background sound exposure reduces sleep-related distress, achieves sound sleep, or improves daytime psychomotor function.

## Introduction

Insomnia is a highly prevalent sleep disorder (Mai and Buysse, 2008). Lack of sleep decreases physical and mental quality of life and work productivity and is also a risk factor for future lifestyle-related diseases, which suggests that improving sleep quality is important for quality of life and prevention of lifestyle-related diseases (Tasali et al., 2009; Rangaraj and Knutson, 2016; Takami et al., 2018).

Various intervention methods, including pharmacotherapy and psychological and behavioral interventions, have been investigated for the treatment of insomnia. However, there are some concerns about the long-term use of medications, such as the risk of abuse, dependence, and adverse effects such as residual daytime sedation, cognitive impairment, and reduced motor co-ordination (Mahfoud et al., 2009). Cognitive-behavioral therapy (CBT-I) is recommended as a first-line treatment for chronic insomnia in adults of any age (Edinger et al., 2021). This treatment provides sustained improvement in sleep and is well accepted by patients (Riemann et al., 2017; Morin et al., 2021). In recent years, music therapy has also been considered a non-invasive intervention to improve sleep in a simple manner (Jespersen et al., 2015).

The influence of music on the structure of sleep is still largely unknown. However, it has been reported that pleasant music has a relaxing effect and reduces anxiety, stress, aversion, cortisol levels, and noradrenaline circulation (Gerra et al., 1998; Lai and Li, 2011; Ventura et al., 2012; Matsuo et al., 2019). It is expected that listening to pleasant sounds during sleep may contribute to improvement of sleep quality.

Several studies have reported on the relationship between music and sleep, and positive effects of music on sleep have been observed in a wide range of age groups (Field, 1999; Tan, 2004; Lai and Good, 2005; Harmat et al., 2008). Previous studies have shown that background music can help preschool children fall asleep faster during naptime (Field, 1999) and improve subjective sleep quality (Tan, 2004). Another study examining the effects of music on young people with poor sleep showed that, while music improved depressive symptoms, it did not improve sleep quality (Harmat et al., 2008). In addition, it has been reported that background music improves subjective sleep quality ratings in older adults as well (Lai and Good, 2005). It has been suggested that music preference is related to the effects of music on sleep (Lai and Good, 2005).

The relationship between music and electroencephalogram (EEG) has been investigated. It is well-known that auditory stimuli containing cyclic changes affect the coherence of EEG (Berger and Turow, 2012). Cordi et al. (2019) composed relaxing music with a frequency of 0.01–2 Hz, which increased slow-wave sleep (SWS) duration.

Sleep spindles in the sleep EEG are waveforms that characterize non-REM sleep Stage 2 (StageN2) (Fernandez and Lüthi, 2020), which play a role in sleep maintenance (De Gennaro and Ferrara, 2003). Sleep spindles have been suggested to be involved in the inhibition of sensory processing during sleep (Steriade et al., 1993; Cote et al., 2000). Cote et al. (2000) investigated changes in event-related potentials (ERP) during sound stimulation of sleeping participants. The amplitude of EPR becomes highest shortly after onset of sound stimulus, which suggests that the spindles may be a consequence of prior thalamic inhibition due to information processing, particularly when being confronted with loud, intrusive, external stimuli (Cote et al., 2000). In addition, sleep spindles are thought to be involved in the maintenance of non-REM sleep continuity (Steriade and Llinás, 1988). In summary, sleep spindles are related to important mechanisms of sleep maintenance.

Sleep spindles have also been reported to be associated with music. Antony and Paller (2017) reported an increase in slow spindles (11–13.5 Hz) when napping participants were exposed to sounds at 11–13.5 Hz, and an increase in fast spindles (13.5–16 Hz) when they were exposed to sounds at 13.5–16 Hz.

Previous studies on the relationship between music and sleep have mainly focused on naps of 45–90 min (Antony and Paller, 2017), while largely ignoring the relationship between music and nighttime sleep. This is because it is burdensome even for a skilled polysomnographic technician to score the EEG for the entire night, particularly to detect spindles (Kinoshita et al., 2020). Thus, in previous studies, the analysis of all-night sleep was limited by the heavy burden on specialists. In this study, we analyzed all-night sleep by using a machine learning algorithm to perform EEG analysis on behalf of specialists.

In this study, we use music similar to the sound of ocean waves (Morishima et al., 2016) as pleasant sounds, which have been confirmed to improve sleep quality. We adopt the automatic spindle detection algorithm developed by Kinoshita et al. (2020) to detect spindles from EEG data, which allows realizing an objective assessment of sleep quality from the viewpoint of spindle occurrence.

The objectives of this study were (1) to investigate whether pleasant sleep sounds shorten sleep onset latency (SOL) and (2) to investigate whether pleasant sleep sounds affect sleep parameters, number of spindles (SN), and spindle density (SD).

## Method

### Trial design

#### Participants

**Figure 1:**
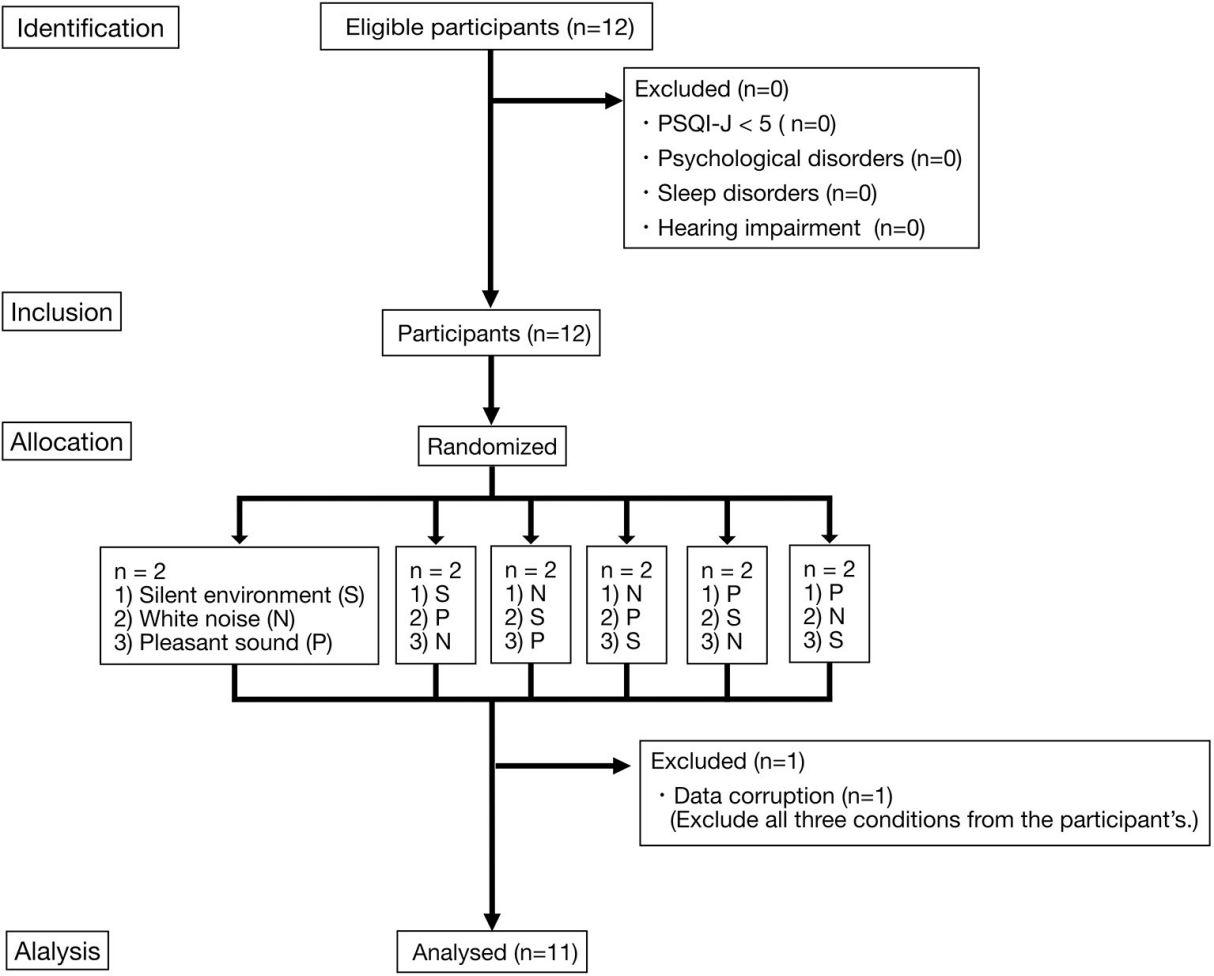
Flow diagram of the participants. Two participants were allocated to each of the three sound environment combinations (six combinations). Each of the three sound environments was played overnight. Each participant took part in the experiment for three nights. Each experiment was conducted at intervals of every 2–3nights. S, Silent environment; N, White noise; P, Pleasant sound.

The study was conducted in Shiga Prefecture, Japan. Japanese participants who were aware of difficulties related to sleep in their daily lives were recruited for the experiment. Participants were recruited between June 24, 2015 and August 28, 2015. Figure 1 shows the flow chart of this study.

Inclusion criteria were as follows: (a) Athens Insomnia Scale (AIS) (Okajima et al., 2020) score ≥ 6, (b) adherence to a controlled sleep schedule, in which participants go to bed at 0:00 a.m. and wake up at 7:00 a.m. for 1 week before the experiment, and (c) not being an employee or a family member of YAMAHA corporation. AIS is a validated 8-item self-report questionnaire that assesses the severity of insomnia with a possible score ranging from 0 to 24 points (Soldatos, 1995). An AIS score of 6 is the optimal cutoff score for insomnia based on a balance of sensitivity and specificity (Soldatos et al., 2003). Cronbach's alpha at pre-assessment and post-assessment was 0.843 and 0.824, respectively.

Exclusion criteria were as follows: (a) those already diagnosed with sleep disorders, psychiatric disorders, or hearing impairment, and (b) no data in questionnaires or polysomnography (PSG). Participants were asked to complete the Japanese version of the Pittsburgh Sleep Quality Index [PSQI (Buysse et al., 1989)], the PSQI-J (Doi et al., 2000), in a pre-test, and participants with a PSQI-J score of 5 or higher were defined as having perceived sleep difficulties. The cutoff score of 5 is the value at which subjective sleep quality is evaluated to be good (Chiu et al., 2016). In a Japanese epidemiological study (Kadotani et al., 2017), about half of the population had PSQI-J < 5. We excluded participants with PSQI-J < 5, because we considered that participants who were unaware of their sleep difficulties would not show any change in their sleep condition even if pleasant sleep sounds were used. The PSQI is an effective self-report questionnaire used to measure sleep quality and patterns in older adults (Buysse et al., 1989). The PSQI distinguishes between “poor” and “good” sleep by measuring seven domains: subjective sleep quality, sleep latency, sleep duration, habitual sleep efficiency, sleep disturbances, use of sleep medication, and daytime dysfunction over the previous month (Buysse et al., 1989). Subjects responded to each item on a Likert Scale of 0 to 3, with 3 corresponding to the negative extreme of the Likert Scale. The PSQI had a Cronbach's alpha of 0.83 for the seven domains.

Next, multiple psychiatrists confirmed that the participants had no psychiatric comorbidities using the Mini-International Neuropsychiatric Interview (M.I.N.I.) (Sheehan et al., 1998), in addition, they confirmed that participants had no diagnosed or currently-treated sleep disorders. Participants were confirmed to have no hearing impairment using an audiometer, AA-77A (RION Co.,Ltd., Tokyo, Japan). No participant satisfied the exclusion criteria before participation. After all experiments were completed, we confirmed that no adverse events had occurred. In this study, follow-up survey was not conducted.

All participants gave written informed consent in accordance with the Declaration of Helsinki. The Ethics Committee of Shiga University of Medical Science approved the study protocol (#26-227). The study was registered at UMIN-CTR (UMIN000019045, Cross-over test for music and sound on sleep, registered on September 16, 2015).

The complete protocol for this study is available upon reasonable request.

#### Details of experiment

PSG was performed in the sleep laboratory at Shiga University of Medical Science. Each participant participated in the experiment every two to three nights. The objective dataset consisted of PSG data (Alice 5, Respironics Inc., Murrysville, PA, USA), for three nights recorded from people during sleep in three types of acoustic environments: silent, white noise, and pleasant sound. Participants arrived at the sleep laboratory at 9:00 p.m., the lights were turned off at 10:00 p.m., and the participants woke up around 6:00 a.m. When exposed to the pleasant sound, they were asked to answer a questionnaire on pleasant sound after arousal (Table 1). The questionnaire on the pleasant sound sleep was answered on a 4-point Likert scale.

**Table 1:** Questionnaire on pleasant sounds.

**Regarding pleasant sounds**	*Answer only for the day when pleasant sounds were played.
No.1 The volume of the pleasant sounds at bedtime was	High ⊢⊣⊢⊣⊢⊣ Low
No.2 The volume of the pleasant sounds at the time of awakening was	High ⊢⊣⊢⊣⊢⊣ Low
No.3 Your impression on the pleasant sounds	Like ⊢⊣⊢⊣⊢⊣ Dislike
No.4 Please state any other opinion or requests regarding the pleasant sound system	

The PSG recordings were from around 10:00 p.m. to 6:00 a.m. the following day. EEG was acquired from Fp1, Fp2, C3, C4, O1, and O2 channels in accordance with the international 10-20 method and recorded by means of referential recording using the earlobe A1 and A2 as reference electrodes.

The details of the order of sound exposure are described below. Two participants were assigned to each of the six possible orders of sound exposure (see Table 2). The sleep laboratory at Shiga University of Medical Science has two PSG rooms, and two different sound conditions were set in advance for each room. The participants were assigned to one of the two rooms by means of the envelope method. The participants were not told which sound would be played on the day of the experiment until the start of the experiment. Due to the characteristics of the experiment, the type of sound that was played during exposure was not concealed from the participants.

**Table 2:** Participant information.

**Participant**	**Age**	**Sex**	**PSQI-J global score**	Order of sound environment*
PART 1	20	Male	5	S-N-P
PART 2	21	Male	8	N-P-S
PART 3	21	Male	8	N-S-P
PART 4	21	Male	5	P-N-S
PART 5	22	Male	8	P-S-N
PART 6	21	Male	6	S-P-N
PART 7	21	Male	9	S-P-N
PART 8	20	Male	9	N-S-P
PART 10	23	Male	7	P-S-N
PART 11	21	Male	6	P-N-S
PART 12	21	Male	8	S-N-P

* S, Silent environment; N, White noise; P, Pleasant sound. All participants were Japanese. Because of data corruption, PART 9 was excluded. PARTs 1, 2, PARTs 3, 4, PARTs 5, 6, PARTs 7, 8, PARTs 9, 10, PARTs 11, 12 were conducted on the same day.

An experienced PSG technologist, who was blind to which background sound was selected in each setting, manually scored sleep stages, and confirmed that none of the participants had abnormal EEG waveforms, such as epileptic discharges.

### Presented sounds

Three types of acoustic environments were used to investigate the effects of sound on sleep quality: white noise, pleasant sounds, and silent, the last one being used as the control. The experimental order of the different acoustic environments was randomly determined for each participant. Pleasant sound, named *Kaiminon* by Yamaha Corporation (Tokyo, Japan), is background sound that is adjusted to oscillate like an ocean wave at a tempo slightly slower than the breathing rate of the person listening to the background sound (Morishima et al., 2016). A previous study has shown that the pleasant sounds used in this study reduce SOL and improve sleep quality in patients with insomnia (Morishima et al., 2016).

The sound was played through speakers in the sleep laboratory overnight. The volume was determined using a sound level meter placed at the position of the participant's head. The silent environment was <30 dB, which is about the sound level of a whisper. The white noise and the pleasant sounds were played at the same volume with an average of 40 dB, which is the same sound level as that of a typical library. The WHO recommends that noise during sleep should be 40 dB or less to prevent negative effects on sleep (World Health Organization., 2018). The volume was determined according to the WHO recommendations in order to avoid negative effects due to sound volume.

### Automatic spindle detection algorithm

Although we investigated effects of sound during sleep on spindles overnight, it is difficult for even skilled polysomnographic technicians to visually label spindles with high accuracy from long-term EEG data. In order to automatically and accurately detect spindles in long-term EEG data, we employed a machine learning-based spindle detection algorithm developed by Kinoshita et al. (2020).

**Figure 2:**
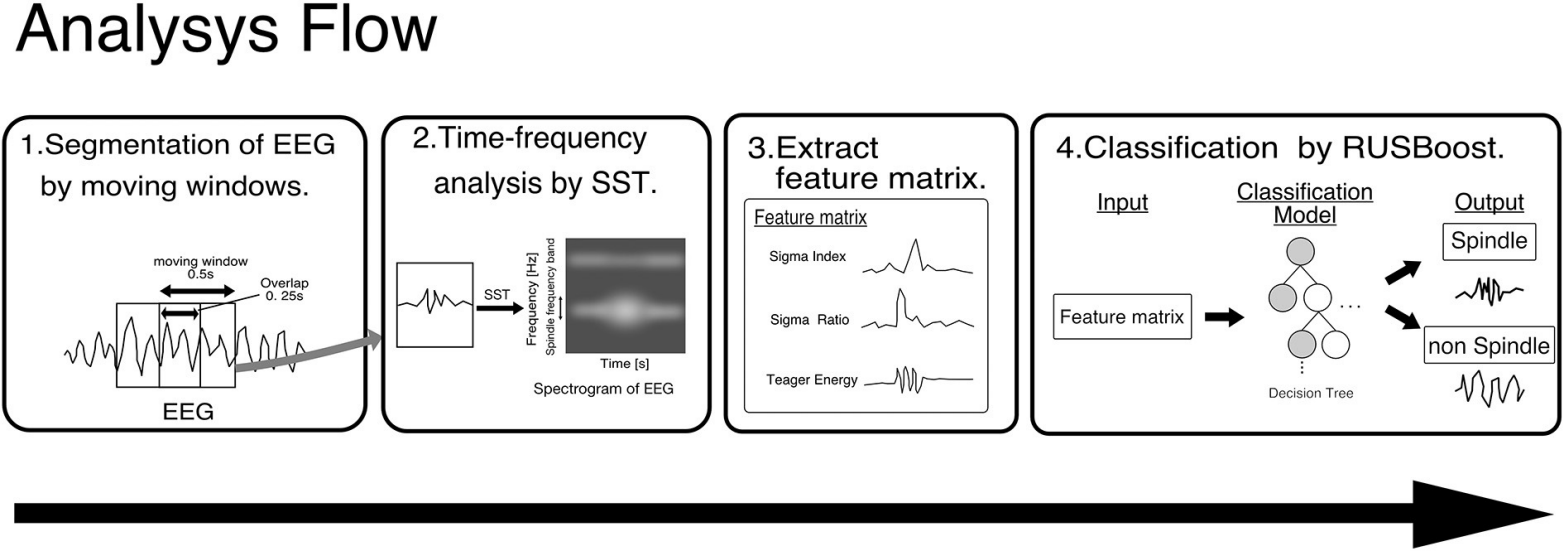
Framework of Kinoshita's algorithm. EEG data are segmented by a moving window of 0.5 s with an overlap of 0.25 s, and SST is applied to the segmented EEG to extract features. The pre-trained classifier detects spindles from the extracted features. SST, wavelet synchrosqueezed transform; RUSBoost, random under-sampling boosting; EEG, Electroencephalography.

Figure 2 shows the flow of Kinoshita's algorithm. In this algorithm, a synchro-squeezed wavelet transform (SST) is applied to the EEG to estimate the frequency of the EEG, and the result is used to create a feature matrix. The small amount of spindle data is then classified using an algorithm called RUSBoost, which is useful for classifying datasets with unbalanced correct labels, such as large amounts of non-spindle data, and spindles are detected. Kinoshita et al. (2020) validated the proposed algorithm using the Montreal archives of sleep studies cohort 1 (MASS-C1), a well-known open PSG dataset. Despite variations in performance when training and validation data were interchanged, Kinoshita et al. (2020) reported an average performance index of 76.9% sensitivity, 61.2% positive predictive value, and F-measure of 0.70, showing higher performance than other spindle detection algorithms.

In this analysis, as in Kinoshita's, spindle detection was performed by analyzing the EEG acquired by PSG with a machine learning model trained using MASS-C1.

### Analysis procedure

We originally planned to evaluate SOL as the primary outcome, and Stage N3 time, WASO frequency, and ECG LF and HF power as secondary outcomes. The spindle detection algorithm, which was developed after the experiment was conducted, enabled us to evaluate spindles as well, so we added SN and SD as outcomes. Other general sleep parameters were also explored as outcomes.

Sleep stages of the collected PSG data were scored from the EEG data in accordance with the AAMS manual, version 2.0 (Berry et al., 2012) by a polysomnographic technician certified by the Japanese Society of Sleep Research. He / she did not know which acoustic environment of the PSG data was being analyzed. In order to evaluate time in bed [min] (TIB), total sleep time [min] (TST), SOL [min], wake after sleep onset [min] (WASO), sleep efficiency [%] (SE), Stage R(%TST), Stage N1(%TST), Stage N2(%TST), Stage N3(%TST) were obtained for each acoustic environment from each participant based on the sleep stage scoring results.

In addition, EEG data measured from the C3 channel during StageN2 were extracted. Sleep spindles were automatically detected using Kinoshita's algorithm. Based on the spindle detection results, StageN2 duration [min] and SD [min^−1^], which is defined as SN [min^−1^], were calculated. Because changes in the training data may affect the spindle detection performance, the mean and standard deviation of the number of spindles were calculated while switching the training data of the spindle detection model ten times. This performance evaluation procedure followed Kinoshita et al. (2020). Since we confirmed that there was no left-right difference in the appearance of spindles in the C3 and C4 channels, only spindles in the C3 channel were used for comparison. Details are described in the Results section.

To investigate the effects of acoustic environment on sleep, sleep parameters (TIB, TST, SOL, WASO, SE, Stage R(%TST), Stage N1(%TST), Stage N2(%TST), Stage N3(%TST), SN, and SD) were compared between two acoustic environment groups (white noise and pleasant sound). The ratios of the sleep parameters between those for environmental noise and silent were compared with the ratios of the parameters between those for pleasant sounds and silent.

Several sleep parameters, including the number of spindles and spindle density, widely vary among individuals. Thus, to investigate changes in sleep parameters in white noise and pleasant sound, we investigated changes in the parameters within individuals compared to silence.

### Subgroup analysis

In a previous study, pleasant sounds shortened SOL in patients with insomnia (Morishima et al., 2016). In view of this result, we focused on healthy people with a tendency of insomnia. WASO of each participant in silent was evaluated, and the five participants with the largest WASO (PARTs 1, 2, 5, 7, and 11) were defined as the sleep maintenance difficulty group. The same parameters were compared among participants in the sleep maintenance difficulty group.

### Statistical analysis

We used the paired-samples *t*-test with a significance level *p* < 0.05 to compare sleep parameters between white noise and pleasant sounds. Effect size (Cohen, 2013) was calculated for comparison of background sound environment on sleep parameters. All computations in this study were performed in MATLAB 2020b (Mathworks Inc.).

We planned a study of a continuous response variable from matched pairs of study participants. Prior data indicate that pleasant sound reduced 40% of SOL. If the difference in the mean response of matched pairs is 600 s with a standard deviation of 500 s, we estimated that we needed a study with 9 pairs of participants to be able to reject the null hypothesis that this response difference is zero with probability (power) 0.8 (PS Power and Sample Size Calculations, ver 3.0, Vanderbilt School of Medicine). The Type I error probability associated with this test of this null hypothesis is 0.05.

## Results

We conducted the experiment on twelve participants who satisfied the participation eligibility criteria. One patient (PART 9) was excluded due to PSG corruption, so that 11 participants were ultimately included in the analysis (21.1 ± 0.83 yrs). The range of PSQI-J global scores was 5–9 points. Table 2 shows the gender, age, PSQI-J global score, and order of exposure of the participants to the sound environment.

Sleep parameters for each participant in the silent environment, white noise, and pleasant sounds are shown in Tables 3–5. Significant differences were not observed between the white noise and the pleasant sounds when comparing the ratios of the changes in the sleep parameters to the silent environment (control) for all participants (*n* = 11). The results of the comparison for all participants are shown in Table 6.

**Table 3:** Sleep parameters with silent environment.

						**Sleep stage (%TST)**				**Sleep spindle**	
**Participant**	**TIB [min]**	**TST [min]**	**SOL [min]**	**WASO [min]**	**SE [%]**	**Stage R**	**Stage N1**	**Stage N2**	**Stage N3**	**SN**	**SD [min^−1^]**
PART 1	458.5	299	19.5	140	65.2	4.5	6.9	77.3	11.4	453 ± 55	1.96 ± 0.24
PART 2	454	279.5	34	140.5	61.6	14.8	10.2	65.7	9.3	297 ± 27	1.62 ± 0.15
PART 3	434	289	86.5	58.5	66.6	5.9	16.8	72.3	5.0	1,336 ± 87	6.39 ± 0.42
PART 4	429	320.5	5	103.5	74.7	21.5	15.4	57.4	5.6	427 ± 35	2.32 ± 0.19
PART 5	435	150	105	179.5	34.5	9.0	26.7	61.7	2.7	428 ± 48	4.63 ± 0.52
PART 6	430	314.5	2	113.5	73.1	14.6	1.7	75.5	8.1	779 ± 58	3.28 ± 0.24
PART 7	435	240	14.5	180.5	55.2	13.3	14.4	66.0	6.3	617 ± 58	3.89 ± 0.36
PART 8	436	320.5	2.5	113	73.5	1.7	12.8	68.0	17.5	592 ± 58	2.71 ± 0.26
PART 10	436.5	352	4	80.5	80.6	13.9	6.8	69.3	9.9	620 ± 62	2.54 ± 0.25
PART 11	430	102.5	71.5	256	23.8	2.9	14.1	75.6	7.3	27 ± 8	0.35 ± 0.11
PART 12	433	296	9	128	68.4	2.9	11.3	70.8	15.0	1,032 ± 67	4.93 ± 0.32
All	437.4 ± 9.7	269.4 ± 76.9	32.1 ± 37.6	135.8 ± 54.4	61.6 ± 17.6	9.6 ± 6.5	12.5 ± 6.5	69.1 ± 6.1	8.9 ± 4.4	600.7 ± 355.3	3.15 ± 1.71

TIB, time in bed; TST, total sleep time; SOL, sleep onset latency; WASO, wake after sleep onset; SE, sleep efficiency; SN, number of spindles; SD, spindle density.

**Table 4:** Sleep parameters with white noise.

						**Sleep stage (%TST)**				**Sleep spindle**	
**Participant**	**TIB [min]**	**TST [min]**	**SOL [min]**	**WASO [min]**	**SE [%]**	**Stage R**	**Stage N1**	**Stage N2**	**Stage N3**	**SN**	**SD [min^−1^]**
PART 1	435	255.5	21.5	160	58.7	1.4	11.2	66.7	20.7	345 ± 38	2.02 ± 0.22
PART 2	443.5	278.5	32.5	131	62.8	11.0	8.6	72.9	7.5	325 ± 35	1.6 ± 0.17
PART 3	435	307	31.5	41.5	70.6	1.6	5.4	83.2	9.8	1,523 ± 104	5.96 ± 0.41
PART 4	431.5	277.5	5.5	149	64.3	16.4	15.0	57.3	11.4	150 ±18	0.94 ± 0.12
PART 5	435	126	144.5	203.5	29.0	0.4	9.9	84.5	5.2	476 ± 45	4.47 ± 0.42
PART 6	430.5	325.5	1	103	75.6	17.2	1.8	70.7	10.3	701 ± 51	3.05 ± 0.22
PART 7	429.5	288.5	11	126.5	67.2	10.1	19.2	68.1	2.6	703 ± 63	3.58 ± 0.32
PART 8	428	217	9	208.5	50.7	4.1	17.3	63.1	15.4	386 ± 35	2.82 ± 0.25
PART 10	437	336.5	15.5	96	77.0	10.3	6.8	74.4	8.5	773 ± 85	3.08 ± 0.34
PART 11	431.5	96.5	104	263.5	22.4	3.6	2.6	84.5	9.3	29 ± 4	0.36 ± 0.05
PART 12	450	304	2.5	136.5	67.6	10.5	6.7	59.4	23.4	852 ± 57	5.39 ± 0.36
All	435.1 ± 6.5	255.7 ± 78.8	34.4 ± 46.5	147.2 ± 61	58.7 ± 18	7.9 ± 6	9.5 ± 5.7	71.3 ± 9.7	11.3 ± 6.3	569.4 ± 410.6	3.02 ± 1.76

TIB, time in bed; TST, total sleep time; SOL, sleep onset latency; WASO, wake after sleep onset; SE, sleep efficiency; SN, number of spindles; SD, spindle density.

**Table 5:** Sleep parameters with pleasant sound.

						**Sleep stage (%TST)**				**Sleep spindle**	
**Participant**	**TIB [min]**	**TST [min]**	**SOL [min]**	**WASO [min]**	**SE [%]**	**Stage R**	**Stage N1**	**Stage N2**	**Stage N3**	**SN**	**SD [min^−1^]**
PART 1	434	309.5	8.5	116	71.3	7.8	15.8	61.9	14.5	404 ± 52	2.11 ± 0.27
PART 2	434.5	271.5	32.5	130.5	62.5	4.4	8.3	79.9	7.4	379 ± 43	1.75 ± 0.20
PART 3	435	326.5	27.5	81	75.1	7.8	13.2	70.0	9.0	1,366 ± 107	5.98 ± 0.47
PART 4	438	267	35.5	135.5	61.0	11.8	23.6	55.4	9.2	195 ± 21	1.32 ± 0.14
PART 5	431	177	33	221	41.1	0.8	27.4	60.5	11.3	378 ± 33	3.53 ± 0.31
PART 6	428.5	336	6	86.5	78.4	15.3	3.6	73.5	7.6	940 ± 74	3.80 ± 0.30
PART 7	434	286.5	6.5	140.5	66.0	10.5	11.2	72.3	6.1	711 ± 66	3.44 ± 0.32
PART 8	434.5	277	10	147	63.8	3.6	9.0	76.7	10.6	526 ± 55	2.48 ± 0.26
PART 10	433.5	352	1	80	81.2	10.1	4.5	73.2	12.2	661 ± 56	2.57 ± 0.22
PART 11	442	98.5	81	262.5	22.3	0.0	6.1	85.3	8.6	98 ± 17	1.17 ± 0.20
PART 12	442.5	275	6.5	161	62.1	8.4	10.4	62.5	18.7	693 ± 45	4.03 ± 0.26
All	435.2 ± 4.2	270.6 ± 73.7	22.5 ± 23.3	142 ± 57.1	62.2 ± 17.1	7.3 ± 4.7	12.1 ± 7.6	70.1 ± 9.1	10.5 ± 3.6	577.4 ± 357.7	2.93 ± 1.41

TIB, time in bed; TST, total sleep time; SOL, sleep onset latency; WASO, wake after sleep onset; SE, sleep efficiency; SN, number of spindles; SD, spindle density.

**Table 6:** Effects of white noise and pleasant sounds on sleep parameters compared to silent environment (All participants *n* = 11).

**Change of sleep parameters**	White noise*[%]	Pleasant sounds*[%]	**Effect size (Cohen's d) pleasant sound-white noise**	***p-values***
TIB	100 (99, 100)	100 (99, 101)	0.013	0.940
TST	96 (86, 103)	100 (95, 110)	0.457	0.119
SOL	110 (63, 142)	72 (38, 207)	0.158	0.683
WASO	107 (92, 117)	103 (88, 128)	−0.089	0.779
SE	96 (88, 103)	102 (92, 111)	−0.044	0.838
Stage R (%TST)	75 (52, 121)	79 (42, 152)	0.459	0.117
Stage N1 (%TST)	97 (48, 120)	81 (74, 128)	0.371	0.215
Stage N2 (%TST)	103 (93, 111)	98 (97, 111)	−0.136	0.677
Stage N3 (%TST)	128 (87, 188)	123 (96, 146)	0.113	0.693
SN	107 (79, 113)	102 (89, 118)	0.382	0.298
SD	99 (93, 103)	94 (85, 108)	0.307	0.423

* Median (first, third quartiles). TIB, time in bed; TST, total sleep time; SOL, sleep onset latency; WASO, wake after sleep onset; SE, sleep efficiency; SN, number of spindles; SD, spindle density.

Table 7 shows the sleep efficiency for each participant in the silent environment. Five participants with large WASO were selected as the sleep maintenance difficulty group. These five subjects also displayed poor sleep efficiency. The same sleep parameters were compared only among the participants in the sleep maintenance difficulty group. The changes in sleep parameters from the silent environment in the sleep maintenance difficulty group are illustrated in Table 8. As a result of the comparisons, significant differences were not found in any of the sleep parameters, even for participants who had difficulty staying asleep. With regard to SOL, pleasant sounds tended to decrease SOL with large effect sizes (*p* = 0.060, Cohen's d = −1.15).

**Table 7:** Sleep efficiency for each participant in silent environment.

Participant*	**WASO [min]**	**Sleep efficiency [%]**
PART 11	256	23.8
PART 7	180.5	55.2
PART 5	179.5	34.5
PART 2	140.5	61.6
PART 1	140	65.2
PART 12	128	68.4
PART 6	113.5	73.1
PART 8	113	73.5
PART 4	103.5	74.7
PART 10	80.5	80.6
PART 3	58.5	66.6

* Listed in descending order of WASO.

**Table 8:** Effects of white noise and pleasant sounds on sleep parameters compared to silent environment (Sleep maintenance difficulty group *n* = 5).

**Change of sleep parameters**	White noise*[%]	Pleasant sounds*[%]	**Effect size (Cohen's d) pleasant sound-white noise**	***p-values***
TIB	98 (98, 100)	99 (96, 100)	0.020	0.931
TST	94 (86, 100)	104 (97, 118)	0.621	0.221
SOL	110 (96, 138)	45 (44, 96)	−1.149	0.060
WASO	103 (93, 113)	93 (83, 103)	−0.129	0.713
SE	94 (90, 102)	109 (102, 119)	0.629	0.235
Stage R (%TST)	74 (30, 75)	30 (9, 79)	−0.051	0.934
Stage N1 (%TST)	85 (37, 134)	81 (78, 103)	0.236	0.438
Stage N2 (%TST)	111 (103, 112)	109 (98, 113)	−0.251	0.574
Stage N3 (%TST)	128 (81, 182)	118 (98, 128)	0.314	0.426
SN	109 (107, 111)	115 (89, 127)	0.509	0.358
SD	99 (97, 102)	108 (88, 108)	0.463	0.400

* Median (first, third quartiles).

**Figure 3:**
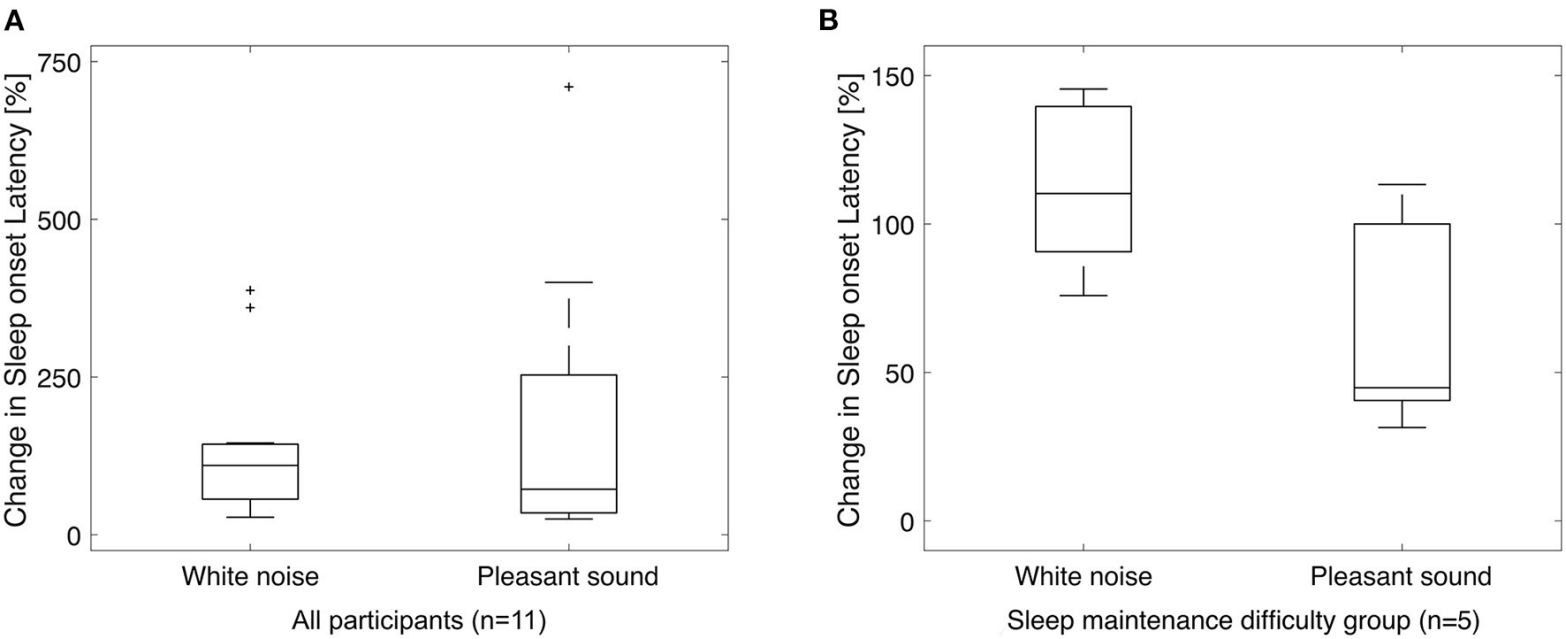
Box-and-whisker plot for sleep onset latency. Sleep onset latency for all participants **(A)** and for the sleep maintenance difficulty group **(B)**. Vertical axis shows the ratio of SOL in the silent environment to 100%. A paired-samples *t*-test (significance level *p* < 0.05, two-sided test) for white noise (left) and pleasant sound (right) showed no significant difference in either case, but there was a significant trend (*p* = 0.060) in the sleep maintenance difficulty group with a large effect size (Cohen's d = −1.15). +, outside values.

Figure 3 shows a boxplot of SOL for all participants and a boxplot for the sleep maintenance difficulty group. SN and SD were not significantly different for the five participants in the sleep maintenance difficulty group overall (Table 8).

Since classifier training and spindle detection were repeated ten times using Kinoshita's algorithm in this study, a *t*-test was conducted based on ten spindle detection results. The number of spindles and the spindle density were compared between each participant (white noise vs. pleasant sounds), which are summarized in Tables 9, 10. A significant increase in spindles was found in seven out of eleven participants (PARTs 1, 2, 4, 6, 7, 8, and 11). A significant increase in the spindle density was found in five out of eleven participants (PARTs 1, 2, 4, 6, and 11).

**Table 9:** Changes in numbers of spindles with white noise and pleasant sounds.

**Participants**		**Number of spindles**		
		**White noise**	**Pleasant sounds**	***p-values***
Sleep maintenance difficulty group	**PART 1**	345 ± 38	404 ± 52	**<0.001**
	**PART 2**	325 ± 35	379 ± 43	**<0.001**
	PART 5	476 ± 45	378 ± 33	<0.001
	**PART 7**	703 ± 63	711 ± 66	**0.033**
	**PART 11**	29 ± 4	98 ±17	**<0.001**
Non-sleep maintenance difficulty group	PART 3	1,523 ± 104	1,366 ± 107	<0.001
	**PART 4**	150 ± 18	195 ± 21	**<0.001**
	**PART 6**	701 ± 51	940 ± 74	**<0.001**
	**PART 8**	386 ± 35	526 ± 55	**<0.001**
	PART 10	773 ± 85	661 ± 56	<0.001
	PART 12	852 ± 57	693 ± 45	<0.001

Participants with increased number of spindles under pleasant sound compared to noise are shown in bold.

**Table 10:** Changes in spindle density with white noise and pleasant sounds.

**Participants**		**Spindle density**		
		**White noise**	**Pleasant sounds**	***p-values***
Sleep maintenance difficulty group	**PART 1**	2.02 ± 0.22	2.11 ± 0.27	**<0.01**
	**PART 2**	1.60 ± 0.17	1.75 ± 0.20	**<0.001**
	PART 5	4.47 ± 0.42	3.53 ± 0.31	<0.001
	PART 7	3.58 ± 0.32	3.44 ± 0.32	<0.001
	**PART 11**	0.36 ± 0.05	1.17 ± 0.20	**<0.001**
Non-sleep maintenance difficulty group	PART 3	5.96 ± 0.41	5.98 ± 0.47	0.706
	**PART 4**	0.94 ± 0.12	1.32 ± 0.14	**<0.001**
	**PART 6**	3.05 ± 0.22	3.80 ± 0.30	**<0.001**
	PART 8	2.82 ± 0.25	2.48 ± 0.26	<0.001
	PART 10	3.08 ± 0.34	2.57 ± 0.22	<0.001
	PART 12	5.39 ± 0.36	4.03 ± 0.26	<0.001

Participants with increased spindle density under pleasant sound compared to noise are shown in bold.

On the other hand, none of the questions in the questionnaire was significant. In other words, pleasant sounds would not improve subjective sleep quality assessment, even if objective sleep quality assessment improved.

**Figure 4:**
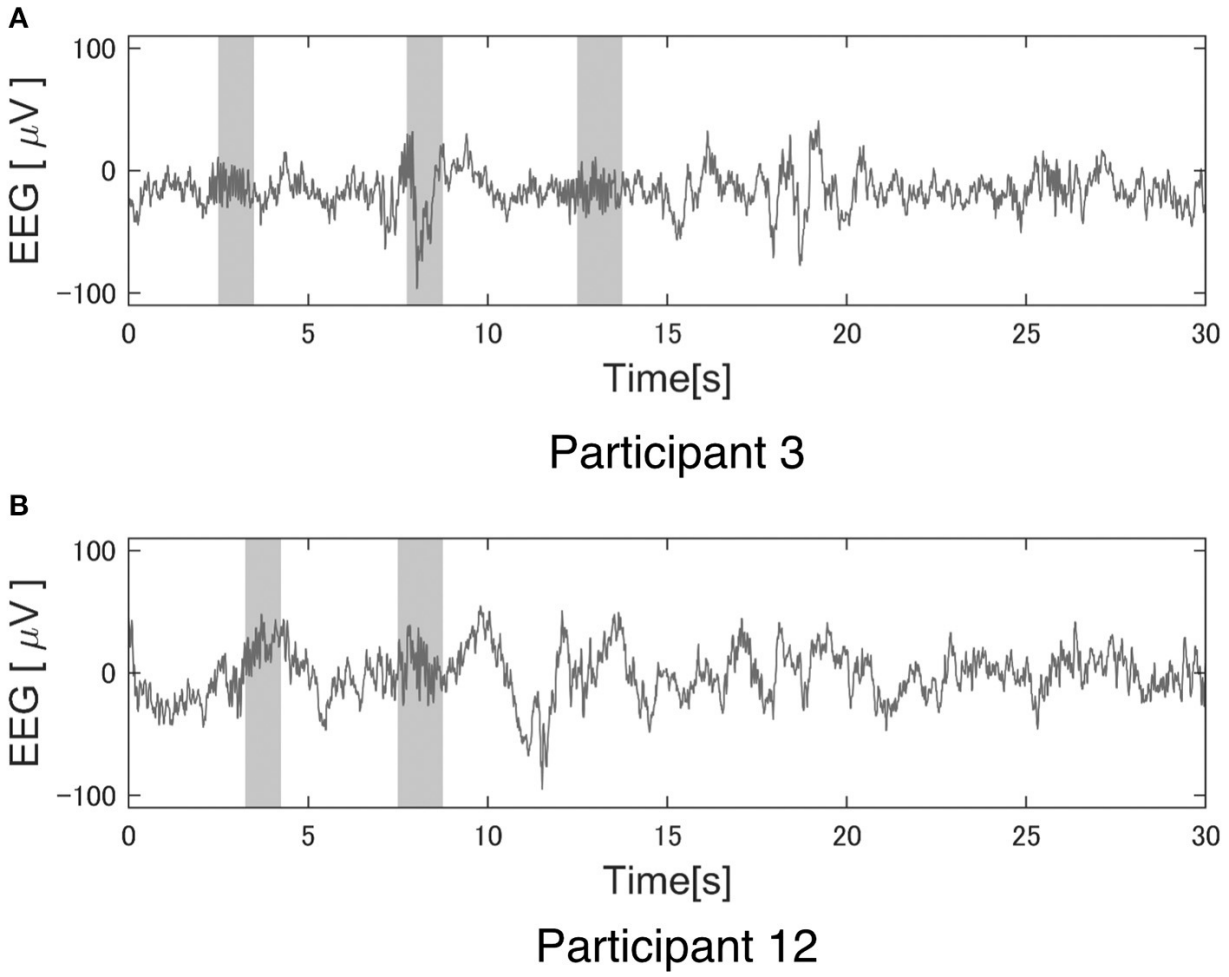
Example of a spindle detection result with Kinoshita's algorithm. Electroencephalography (EEG) data were clipped from the C3 channel for 30 s during StageN2: **(A)** Participant 3 under the pleasant acoustic environment and **(B)** Participant 12 under white noise. The colored bands are the detected spindles. These figures clearly show that Kinoshita's algorithm appropriately detected spindles.

The reliability of this study largely depends on the performance of Kinoshita's algorithm. Figure 4 shows an example of Kinoshita's algorithm applied to EEG data. The colored bands in Figure 4 are the spindles detected by the algorithm, clearly showing that the detection result is consistent with the visual detection of spindles by the polysomnographic technician. Thus, we confirmed that Kinoshita's algorithm has adequate performance for spindle detection.

**Figure 5:**
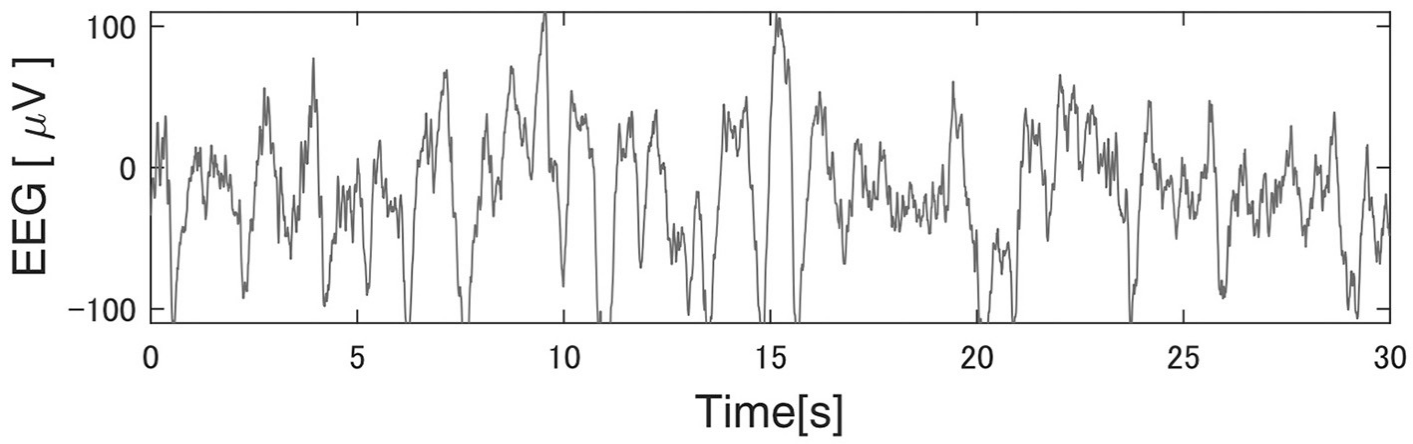
Typical EEG data of the C3 channel in Participant 11. Kinoshita's algorithm could not correctly detect the spindles, because this participant's electroencephalography (EEG) data had significant artifacts. He had extremely poor sleep efficiency in comparison with other participants, suggesting that the artifacts were generated by body movement.

Tables 3–5 show that PART 11 had a notably smaller number of spindles in comparison with the other participants. Figure 5 shows his EEG data at the C3-A2 channel in sleep StageN2. The EEG and video of PART 11 showed body motion. These results suggest that PART 11's EEG data contains many body movement artifacts, which may have contributed to the spindle detection. Table 7 shows that PART 11 also had a notably low sleep efficiency, which may imply the presence of significant body motions during the participant's sleep. We should be careful when applying Kinoshita's algorithm when the objective EEG data contain significant artifacts.

Spindles were detected from the C3 channels in this study. The number of spindles is considered to be almost the same between the left and right channels (Ebersole and Pedley, 2003). We applied Kinoshita's algorithm to the C4 channel in order to check the laterality of the spindles detected by Kinoshita's algorithm. We confirmed that there was no laterality in the detected number of spindles by means of a paired-samples *t*-test (C3:601 ± 339 vs. C4:517 ± 289, *p* = 0.0510). Thus, it was appropriate to perform analysis only on the C3 channel.

## Discussion

In this study, we investigated the effects of three types of acoustic environments on overnight sleep. The results showed that pleasant sleep sounds did not have a statistically significant effect on sleep status in any of the participants, even when limited to those who had sleep maintenance difficulty. Pleasant sounds tended to shorten SOL with large effect sizes compared to white noise (*p* = 0.060, Cohen's d = −1.15). In a previous study, a significant reduction in SOL was observed in patients with insomnia exposed to pleasant sounds (Morishima et al., 2016), and although the results of the present study were not consistent with this finding, a trend toward a reduction in SOL was observed. In this study, the number of participants recruited was determined based on the expectation that pleasant sounds would shorten SOL by 40%. However, pleasant sounds had no expected effect on shortening SOL, and the small sample size may be another reason why no significant differences in sleep parameters were observed.

We confirmed that the pleasant sounds did not improve subjective sleep quality. This is not consistent with results of previous studies that investigated the relationship between sound and subjective sleep quality (Field, 1999; Harmat et al., 2008; Jespersen et al., 2015). According to a result of the questionnaire about pleasant sounds shown in Table 1, five out of eleven participants gave a score of third in the four-point scale for the pleasant sounds in item No. 3 “Your impression on the pleasant sounds.” The other six participants gave a score of second. This suggests that the participants may not have had a good impression of the pleasant sounds. It has been reported that the effect of music on emotions is also related to the individual's music preferences (Field, 1999; Tan, 2004; Lai and Good, 2005; Harmat et al., 2008). PARTs 1, 2, 7, and 11 belonged to the sleep maintenance difficulty group. This suggests that the pleasant sounds affect sleep, at least in terms of spindles, especially in participants who have difficulty maintaining sleep. Despite the fact that the results of the questionnaire showed that the pleasant sound was not a favorite, some participants showed a change in the SN and SD, suggesting that background sound environment may influence brain activity during sleep, regardless of the preference for background sound.

When considering the application of music intervention to sleep, the lack of a subjective sleep quality assessment may be disadvantageous in terms of motivating treatment. In order to consider individual preferences, music used for therapy should be selected from various musical categories.

This study was conducted based on a method that was developed after the experiment was conducted, so there is a limitation in the interpretation of the results shown. Limitations of this study include the collected data, such as all the participants being Japanese and healthy young males; that is, we could not consider racial, sex, or age differences in this study. We did not record AIS to confirm the inclusion criteria. Instead, we used the PSQI-J to confirm the subjective sleep quality of the participants. We used a cutoff score of 5 on the PSQI-J to select participants with sleep problems (as described in the methods section). However, it is a limitation that we did not use a score of 5.5, which is the usual cutoff for PSQI-J (Doi et al., 2000). The trend toward shorter SOL suggests that pleasant sounds may have some effect on improving sleep quality in a non-invasive manner in people who are aware of their insomnia. We need to collect additional data from a variety of people to confirm our results. In addition, it is noteworthy that our analysis results cannot be directly applied to insomnia patients because they may be using sleeping pills, which could affect sleep quality and spindles. Another limitation was that only one type of pleasant sound was used in the experiment. This limitation did not allow us to determine the influence of the sleep index on subjective music preference.

The protocol and data that support the findings of this study are available from the corresponding author upon reasonable request.

We confirmed a significant increase in spindles under the pleasant acoustic environment in seven out of eleven participants as compared with white noise. In addition, there was a trend toward a decrease in SOL in the sleep maintenance difficulty group. The results suggest that pleasant sounds may have some effect on sleep for participants with large WASO. Further research is needed to determine whether background sound exposure reduces sleep-related distress, produces a sense of sound sleep, or improves psychomotor function.

## Conclusion

In this study, we conducted PSG with eleven healthy young male participants under three types of acoustic environments: silent, white noise, and pleasant sounds. We detected spindles from the EEG data recorded by PSG with an automatic spindle detection algorithm and evaluated the spindles and other sleep parameters.

While this study was conducted on healthy young participants, future studies will be conducted on patients with insomnia. In addition, we will test an intervention method for insomnia that combines music therapy with pharmacotherapy or CBT-I. In this study, the effect of pleasant sound on hyperarousal, which often occurs in patients with insomnia, is unknown, and thus is worth further investigation.

## Data availability statement

The raw data supporting the conclusions of this article will be made available by the authors, without undue reservation.

## Ethics statement

The studies involving human participants were reviewed and approved by the Ethics Committee of Shiga University of Medical Science. The patients/participants provided their written informed consent to participate in this study.

## Author contributions

YS, KF, MM, and HK conceived the idea. YS, MM, and HK performed the experiments, interpreted the data, and revised the manuscript. YS and KF analyzed data. TK contributed to the materials and the music used in this study. SS and KF wrote the draft. All authors contributed to the article and approved the submitted version.

## Funding

This study received funding from Yamaha Corporation. The funder was involved partially in the study design, but the funder was not involved in the collection, analysis, and interpretation of data, the writing of this article, or the decision to submit it for publication.

## Conflict of interest

The data in this study were obtained in a joint research project between Shiga University of Medical Science and Yamaha Corporation. This study received funding from Yamaha Corporation. The funder was involved partially in the study design, but the funder was not involved in the collection, analysis, and interpretation of data, the writing of this article, or the decision to submit it for publication. KF is employed by Quadlytics Inc. HK received grants from the Investigator-Initiated Studies Program of Merck Sharp and Dohme LLC/MSD K.K., Eisai Co., Ltd. HK reports consulting fees from Takeda Pharmaceutical Co. Ltd. HK was supported by donations from FukudaLifetech Co., Ltd. and Fukuda Life Tech Keiji Co., Ltd. KY and TKa were employed by Yamaha Corporation. Dohme LLC/MSD K.K., Eisai Co. Ltd., Fukuda Life Tech Co. Ltd., and Fukuda Life Tech Keiji Co. Ltd. had no role in the design of the study; in the collection, analyses, or interpretation of data; in the writing of the manuscript, or in the decision to publish the results. The remaining authors declare that the research was conducted in the absence of any commercial or financial relationships that could be construed as a potential conflict of interest.

## Publisher's note

All claims expressed in this article are solely those of the authors and do not necessarily represent those of their affiliated organizations, or those of the publisher, the editors and the reviewers. Any product that may be evaluated in this article, or claim that may be made by its manufacturer, is not guaranteed or endorsed by the publisher.

## Abbreviations

CBT-I: Cognitive behavioral therapy
EEG: electroencephalogram
ECG: electrocardiogram
SWS: slow-wave sleep
PSG: polysomnography
WHO: World Health Organization
SST: synchro-squeezed wavelet transforms
RUSBoost: random under-sampling boosting
MASS-C1: Montreal archives of sleep studies cohort 1
PART: Participant
TIB: Time in bed
TST: Total sleep time
SOL: Sleep onset latency
WASO: Wake after sleep onset
SE: Sleep efficiency
Stage R: REM sleep
Stage N1: non-REM sleep stage 1
Stage N2: non-REM sleep stage 2
Stage N3: non-REM sleep stage 3
SN: Number of spindles
SD: Spindle density.
